# Honour across borders: How cultural norms shape prejudice confrontation in migration contexts

**DOI:** 10.1111/bjso.70034

**Published:** 2025-12-17

**Authors:** Mete Sefa Uysal, Thomas Kessler, Ayse K. Uskul

**Affiliations:** ^1^ Department of Psychology University of Exeter Exeter UK; ^2^ Department of Social Psychology Friedrich‐Schiller University Jena Jena Germany; ^3^ School of Psychology University of Sussex Brighton and Hove UK

**Keywords:** culture, discrimination, honour, prejudice confrontation, retaliation

## Abstract

How do internalized cultural values shape responses to discrimination among minoritized groups? This research investigates how honour values, originating from socio‐ecological contexts marked by insecurity and weak institutional protection, shape prejudice confrontation among individuals from honour‐culture backgrounds living in Western European dignity cultures. Across three studies, we examined South and West Asians in the United Kingdom and Turkish post‐migrants in Germany. We tested whether endorsement of collective honour and modern proxies of socio‐ecological conditions in which honour cultures emerge (e.g., perceived financial threat, low trust in police effectiveness and procedural unfairness) predict intentions to confront discrimination. Studies 1 and 2 showed that the frequency of discrimination experiences and collective honour predicted aggressive confrontation. Studies 2 and 3 showed the dual role of honour norms: endorsement of honour norms related to family reputation predicted only non‐aggressive confrontation, whereas endorsement of retaliation norms predicted only aggressive confrontation. Study 3, a pre‐registered experiment, found no causal effect of manipulated contemporary manifestations of long‐term socio‐ecological conditions on honour endorsement or confrontation. Together, findings suggested that lived experiences of discrimination, alongside honour norms, predict confrontation. Moreover, they highlight the importance of distinguishing between dimensions of honour norms when examining culturally grounded responses to intergroup discrimination.

## INTRODUCTION

“Where did they think they were living? They were behaving in a way that wasn't familiar even in the poems that I studied in school, in the novels I read. I was puzzled. They weren't reacting to the insults.

My Brilliant Friend, Elena Ferrante.”

This quote captures Lenu's astonishment at Stefano, her friend Lina's fiancé, who, living in 1950s Naples, fails to respond to gossip and insults about Lina's fidelity. Though fictional, this quote vividly illustrates the power of cultural expectations and the social consequences of defying them. This is especially true when cultural expectations determine one's place and reputation in society. In contexts where reputation is collectively held and public judgement carries weight, failing to respond to affronts can signal weakness or dishonour (Cohen et al., 1996; Peristiany, 1965; Uskul et al., 2012). When one's value and status are dependent on others' perceptions, and when those perceptions can be publicly challenged, protection of one's image by confronting insults becomes not just expected but required (Cohen & Nisbett, 1994; Cross et al., 2013; Uskul et al., 2015; van Osch et al., 2013).

It is no coincidence that this example comes from Southern Italy, where honour is a strong determinant of one's value and status in society, and where any insult to these requires immediate confrontation and reparation (Uskul et al., 2023; Vignoles et al., 2024). Building on this cultural logic, we argue that such internalized cultural expectations can shape one's reactions to insults, especially in intercultural contexts where the victim of the insult is residing in another culture than their own and has different cultural scripts from the perpetrators. In this paper, we explore the extent to which individuals from honour‐culture backgrounds—who have moved to non‐honour cultural contexts or socialize as post‐migrants in these contexts—internalize honour values, and how these internalized values shape their responses to discrimination in societies where their group is minoritized and where their cultural identity is often threatened or devalued. Across two correlational and one pre‐registered experimental study, we examined the extent to which endorsement of honour values and socio‐ecological conditions historically linked to the development of honour cultures—perceived financial threat, low trust in police effectiveness and low procedural fairness—predict intentions to confront discrimination in different forms. We focused on individuals from honour‐culture backgrounds living in Western European dignity cultures: South and West Asians in the United Kingdom, and the Turkish post‐migrants in Germany.

### Prejudice confrontation

Prejudice confrontation, defined as verbally or non‐verbally expressing dissatisfaction with biased or discriminatory behaviour, has been widely recognized as an effective strategy to reduce future prejudice and stereotyping by confronted perpetrators (Ashburn‐Nardo et al., 2008; Barreto, 2015; Barreto & Ellemers, 2015; Chaney et al., 2015; Shelton et al., 2006). For instance, white participants who were confronted for using racial stereotypes showed reduced use of such stereotypes a week later compared with those who were not confronted (Chaney et al., 2021; Chaney & Sanchez, 2018). Moreover, confronting prejudice not only curbs individual bias but also disrupts the validation of discriminatory social norms, which otherwise normalizes bias and renders it invisible to observers (Blanchard et al., 1994; Chaney & Sanchez, 2022; Czopp, 2019). Therefore, confrontation serves both as a corrective tool and as a vehicle for promoting egalitarian norms across social contexts (Gulker et al., 2013).

However, promoting social change or reducing prejudice is not always the only motivation behind confrontation (Becker & Barreto, 2019). For instance, research on stigma‐induced threat and racism‐related stress (e.g., Harrell, 2000; Major & O'Brien, 2005; Mellor, 2004) has examined confrontation as a coping strategy against racism and stigma. More recently, Chaney et al. (2015) argue that confrontation serves as a coping strategy for targets of discrimination, contributing to psychological outcomes such as greater life satisfaction and a sense of autonomy. For instance, women who publicly confronted a sexist article via a tweet reported experiencing less negative affect than those who did not (Foster, 2015). Frequent confrontations of prejudice are also associated with reduced anger and regret, and increased feelings of empowerment, autonomy and closure (e.g., Gervais et al., 2010; Hyers, 2007; Uysal & Acar, 2025). Thus, confronting prejudice may promote psychological well‐being for the confronter (Chaney et al., 2015; Foster, 2013).

Yet, this body of work has primarily focused on its effectiveness in managing stigma and racism and its consequences for health and well‐being. Relatively little research has explored *why* and *how* individuals choose to confront prejudice. Among the limited studies addressing the *how*, various classifications have emerged: low‐threat versus high‐threat confrontations (Czopp et al., 2006); assertive versus unassertive approaches (Dickter et al., 2012); aggressive versus non‐aggressive styles (Becker & Barreto, 2014); and strategies categorized as angry, educational, or indirect (Foster, 2013), as well as educational, argumentative, help‐seeking, empathetic and humorous (Chaney & Sanchez, 2022). While recent work has begun to go one step further from prejudice reduction and examine the consequences of different confrontation styles on confronters, such as perceived effectiveness, emotional outcomes like rumination and the sense of autonomy (Chaney et al., 2021; Chaney & Sanchez, 2018, 2022), the underlying motivations for confronting prejudice remain relatively underexplored. According to Becker and Barreto (2019), individuals may be driven by three main types of motives: personal motives, which involve a desire to stop prejudicial treatment directed at oneself; collective motives, which aim to address group‐based discrimination and improve conditions for the broader group (see also Uysal et al., 2024, Uysal, Drury et al., 2025; Uysal, Martinez, et al., 2025 on confrontational collective action); and group‐distancing motives, which reflect an effort to disassociate oneself from the stigmatized group, aligning with the concept of individual mobility (Tajfel & Turner, 1979).

In addition to the limited number of studies on predictors of prejudice confrontation, a recent meta‐analysis showed that most studies on prejudice confrontation have been conducted in artificial settings, primarily with the U.S.‐based, young, white participants, and largely focused on whether confrontation reduces stereotype use and improves well‐being (Wood et al., 2025). As a result, cultural dimensions of prejudice confrontation are often overlooked. What is considered beneficial, or what motivates a target to confront, is frequently examined through a singular, often Eurocentric lens.

Even when we extend our scope to include research on intergroup harmony and conflict resolution involving participants from the majority world (also known as *non‐WEIRD* or *Global South*, for example, Africa, Asia and South America; see Uskul et al., 2024), a critical limitation remains, one that prevents us from achieving a broader understanding of the motives behind confrontation. In studies of intergroup harmony and social cohesion, social psychology typically approaches these issues within the framework of national borders and two conflicting ethno‐national identities (for discussion, see Dixon et al., 2020). In other words, it tends to focus on the question of how groups perceived as the ‘rightful owners’ of a land (e.g., majorities, host cultures), according to nation‐state logics or ethno‐national narratives, can coexist with a group seen as outsiders or late arrivals (e.g., minorities, refugees). While this line of inquiry is undoubtedly important, it also reflects a form of reductionism. The ethno‐national identities of these groups constitute only one layer of the challenges involved in living together and achieving cohesion. National identity is a relatively recent construct in human history, and although the tensions it produces are central, they are not sufficient to explain the full complexity of intergroup dynamics. Human behaviour, emotion and cognition have been shaped by a long cultural‐evolutionary process (Henrich, 2017; Richerson & Boyd, 2006), and relying solely on national identities that emerged in the last couple of centuries to explain this vast psychological landscape is deeply limiting.

To gain a fuller understanding, we must look to the longer arc of cultural evolution. If we consider the cultural scripts shaped by the socio‐cultural and ecological environments in which people live, we can better understand behavioural patterns, especially when those patterns differ not only by group identity but by deeply ingrained ways of being. These cultural behaviour scripts not only prescribe what behaviours are considered appropriate in a given context, but also anticipate the likely responses or counter‐behaviours that will follow. When individuals from different groups, each guided by distinct cultural scripts, interact, these expectations may collide. The result is a disruption of the distinct cultural scripts, where mutual misunderstandings and conflicting behavioural norms can give rise to tensions or conflicts. Therefore, we argue that cultural psychology, particularly the psychology of honour cultures, offers a vital theoretical lens for explaining the motivations behind confronting discrimination, especially in contexts where minority communities with honour‐culture backgrounds coexist with majority groups rooted in dignity cultures, as is the case in much of the United Kingdom and mainland Europe.

### What can we learn from the psychology of honour cultures?

Honour plays a central role in shaping both the self‐concept and social interactions in Latin America, North Africa, South and West Asia and the southern United States, which cultural psychologists regard as honour cultures (for a review, see Uskul et al., 2019). Unlike dignity cultures, where self‐worth is overwhelmingly independent of others' judgements and social relationships, self‐worth in honour cultures is more context‐dependent, more often subject to social validation, and hence, in constant need of proof, defence and demonstration (Leung & Cohen, 2011). In these contexts, maintaining or restoring honour often requires projecting a reputation for toughness and signalling a readiness to retaliate against perceived threats or insults to protect oneself and one's family (Uskul et al., 2023; Uskul & Cross, 2019). As such, individuals' social standing and reputations are maintained through visible demonstrations of strength, retributive action and the proactive defence of both personal and collective honour (Leung & Cohen, 2011).

These cultural scripts, which emphasize retaliation as a means of safeguarding reputation and status, have been linked to distinctive patterns of aggressive behaviours in honour cultures. For example, the southern United States, considered an honour culture, has historically shown higher rates of argument‐related homicides compared with the northern United States, considered a dignity culture. Cohen and Nisbett (1994) proposed that this disparity may be partially attributed to an honour ideology that legitimizes conflict when it serves to restore one's honour through retaliation. Importantly, their findings indicate that Southerners justify conflict as self‐defence or in response to honour threats, rather than a general endorsement of violence or conflict.

Building on this framework, subsequent research in social and cultural psychology has documented how honour‐motivated behaviours often emerge in the form of defensive aggression during interpersonal conflicts, such as insults or affronts (e.g., Cohen & Nisbett, 1997; Cohen et al., 1996; Uskul et al., 2012, 2015; for a review see Uskul & Cross, 2019). Consistently, studies have found that individuals from honour cultures tend to react more aggressively to insults and are more likely to endorse confrontation or retaliation than individuals from dignity cultures (Cohen et al., 1996; Uskul et al., 2012, 2015). Yet, although honour is often associated with aggression and violence, the cultural logic of honour and individuals' endorsement of honour codes is fundamentally about confrontation, not aggression. Socialization in honour cultures encourages individuals to internalize dominant norms that obligate them to respond to violations. Maitner et al. (2022), for instance, showed that when perpetrators took resources from victims, participants from dignity and face cultures were more likely to expect that the victim should do nothing, whereas participants from honour cultures reported higher expectations that the victim should confront.

Scholars also examine the socio‐ecological factors that facilitate the emergence of honour cultures. Honour norms tend to emerge in contexts where economic deprivation and uncertainty intersect with intergroup conflict and theft, particularly when such acts are not adequately or fairly addressed by legal authorities (e.g., Barnes et al., 2014; Brown & Osterman, 2012; Nisbett & Cohen, 1996). In these environments, cultural norms emphasize intolerance of provocations directed at the self, family, clan, tribe, national ingroup or property, because honour codes serve a survival function in settings where law enforcement is weak or unreliable, and where resources are scarce or contested (Altheimer, 2012; Nowak et al., 2015; Uskul & Cross, 2020). Early research on honour cultures further shows that these norms developed under socio‐ecological pressures that fostered the formation of tight‐knit familial clans or tribes, which functioned as the primary unit of social protection and collective strength against external threats (Fischer, 1989; Pitt‐Rivers, 1966).

We argue that ethnic or national identities may partly serve as modern extensions of this tribal mentality and clannishness. Individuals who endorse honour values are especially likely to view their national identity as central to their self‐concept and to invest significant emotional and symbolic meaning in it (Barnes et al., 2014; Maitner et al., 2017). Research shows that people from honour cultures report stronger anger in response to insults targeting their national identity than to those directed at other social identities, such as student identity (Maitner et al., 2017). Through affiliation with a cultural group that others are expected to think twice before provoking, honour endorsers may be enacting a time‐tested strategy of using collective identity as a protective shield against harm (Barnes et al., 2014). Hence, aggressive forms of prejudice confrontation may be understood as a manifestation of this social‐evolutionary legacy, reactivated in contemporary settings by perceived economic insecurity and inadequate institutional protection. This is particularly relevant in Western European societies, where individuals from honour cultures often constitute marginalized communities with migration backgrounds. In other words, the survival codes embedded in the socio‐environmental conditions that originally shaped honour cultures can be reawakened in modern intergroup relations when individuals face deprivation, injustice, or discrimination related to their culture. Under such conditions, protective honour norms may become activated, increasing the likelihood of aggressive confrontation against discrimination among minorities with honour‐culture backgrounds in the Global North through the strong endorsement of collective honour in the form of defensive ethnic honour.

### Current research and hypotheses

The present research investigates how contemporary socio‐ecological conditions may predict cultural norms of honour and shape responses to discrimination, particularly among minoritized groups from honour‐culture backgrounds living in Western European dignity cultures. Drawing on cultural psychology and socio‐ecological theories of honour, we examine whether perceived financial threat and low trust in legal authorities—modern indicators of conditions historically associated with the emergence of honour norms—predict endorsement of collective honour and, in turn, prejudice confrontation. Specifically, we hypothesize that financial threat will positively predict endorsement of collective honour and the likelihood of engaging in prejudice confrontation. In addition, trust in police effectiveness and procedural fairness will negatively predict collective honour endorsement and prejudice confrontation. We hypothesize that the endorsement of collective honour will predict prejudice confrontation, whether expressed in aggressive or non‐aggressive forms. In this research, we used aggressive confrontation as a broad term for actions in response to prejudice and discrimination, intended to harm perpetrators of discrimination, whether through physical violence, verbal attacks, or psychological intimidation. The frequency of experiencing discrimination is included as a covariate in the model for its potential correlation with collective honour endorsement and prejudice confrontation. We tested these hypotheses in two contexts where honour‐culture‐background minoritized groups live in countries where majorities do not endorse honour norms: South and West Asians in the United Kingdom (Study 1) and Turkish post‐migrants in Germany (Studies 2 and 3).

## STUDY 1

### Method

#### Participants and procedure

We collected data from participants who live in the United Kingdom and have South and West Asian backgrounds, exemplifying honour cultures. To ensure that our participants are of honour cultural backgrounds, we pre‐screened our participants from a pool of 735 participants using Prolific one week before starting actual data collection. In the pre‐screen phase, participants were asked, ‘*In the UK, independent of whether they identify as a British national or not, many people would also consider having an ethnic or cultural background (e.g., Welsh, Pakistani, Italian). Which of the countries below describes your ethnic/cultural background best?*’ We then targeted 400 participants whose cultural backgrounds are honour cultures such as India, Pakistan, Bangladesh, Sri Lanka, Iran, Turkey and Nepal. Data were collected in December 2022. A total of *n* = 398 participants were recruited. After removing 17 participants who did not complete the survey, the final sample consisted of *n* = 381 participants (185 males, 193 females and 3 did not prefer to say). Participants' ages ranged from 18 to 77 (*M* = 30.31, *SD* = 9.70). Most had a graduate or higher degree (70.3%). From 0 = left (liberal) to 7 = right (conservative), on average, participants were left‐leaning (*M* = 2.88, *SD* = 1.39). In terms of religiosity, on a response scale from 0 = *I have no religious beliefs* to 7 = *I am as religious as I can be*, participants were moderate on average (*M* = 4.32, *SD* = 2.12).

#### Measures

We used 5‐point response scales (1 = *strongly disagree* to 5 = *strongly agree*) unless otherwise stated.

##### Prejudice confrontation

Participants' willingness to confront discrimination is assessed by 11 items: 10 items adapted from the Prejudice Confrontation Styles Scale (Chaney & Sanchez, 2022) and an additional item created for the current study. Participants rated the different confrontation styles that they might use when someone discriminates against them based on their ethnic or cultural background (1 = *not at all true for me* to 5 = *very true for me*). Although the original scale suggested four factors (argumentative, educational, empathy and humour), principal factor analysis in the current study showed that argumentative and empathy items loaded into the same factor, resulting in three distinct factors: aggressive confrontation, non‐aggressive confrontation and avoidance (see Supplementary Material S1, pp. 1). Accordingly, avoidance was measured with two items (humour factor in the original scale): ‘*I make a joke about it and hope they understand I disagree*’ and ‘*I kid around about their ignorance*’ (*r* = .60). We measured non‐aggressive confrontation with five items (educational and empathy in the original scale): ‘*I educate them about the negative impact of discrimination*’, ‘*I show them why what they said was discriminatory*’, ‘*I make sure they know I am saddened by what they said*’, ‘*I help them be better able to spot discrimination*’ and ‘*I tell them I am upset by what they said*’ (α = .87). Finally, aggressive confrontation was assessed by four items (argumentative factor in the original scale and an additional item that was created by researchers): ‘*I threaten to beat them*’, ‘I *dominate the discussion and don't let them get a word in*’, ‘*I talk louder than them so I can't be interrupted*’ and ‘*I slap them*’ (α = .76).

##### Endorsement of collective honour

We created four items to measure the endorsement of collective honour. The items were ‘*Being a member of my ethnic group is something honourable to me*’, ‘*I must stand strong against the people who insult my ethnic group*’, ‘*To maintain my honour, I must not let others insult my ethnic group*’ and ‘*I must always defend my ethnic group's reputation*’ (α = .85).

##### Financial threat

To measure the extent to which participants experienced economic threat, we used five items adapted from Marjanovic et al. (2013). Participants indicated how they feel about their current financial situation by answering the following questions (1 = *not at all*, 5 = *great deal*): ‘*How uncertain do you feel?*’ ‘*How much do you feel at risk?*’ ‘*How much do you think about it?*’ ‘*How much do you worry about it?*’ and ‘*How much do you feel threatened?*’ (α = .91).

##### Trust in police effectiveness

Participants' trust in law enforcement was measured using four items adapted from Akinlabi and Murphy (2018). Participants rated items by considering the police force in their city or town. Items were: ‘*The police respond promptly to calls about crime*’, ‘*The police are always ready to provide satisfactory assistance to victims of crime*’, ‘*The police are doing well at controlling violent crime*’ and ‘*Overall, the police are doing a good job in my neighbourhood*’ (α = .91).

##### Procedural fairness

We measured the perceived procedural fairness justice system using three items adapted from Messing et al. (2015). The items were: *How much confidence do you have that* ‘*the police in the UK will not use excessive force on suspects from your ethnic group*’, ‘*police officers in the UK will treat your ethnic group fairly*’ and ‘*the courts in the UK will treat your ethnic group fairly*’ (1 = *very little*, 4 = *great deal*; α = .91).

##### Discrimination experiences

We assessed how often, during the last year, participants experienced various forms of discrimination because of their ethnic group on a scale from 1 (never) to 5 (daily) using five items adapted from Good et al. (2012). The sample item reads, ‘*How often have you been insulted or called a name because of your ethnic background*’ (α = .91).

### Results

First, we conducted preliminary analyses to examine the relations between variables. Correlations among the variables and descriptive statistics are presented in Table 1.

**TABLE 1:** Means, standard deviations and correlations among variables, Study 1.

Variables	*M* (*SD*)	1	2	3	4	5	6	7	8
1. Aggressive confrontation	1.55 (.66)	–	.24***	.27***	.13**	.04	.02	−.02	.20***
2. Non‐aggressive confrontation	3.17 (.98)		–	.13*	.35***	.08	−.02	−.14**	.16**
3. Avoidance	2.59 (1.10)			–	−.02	−.03	−.02	−.04	.07
4. Collective honour	3.78 (.86)				–	.08	.03	−.08	.06
5. Financial threat	3.09 (.99)					–	−.17**	−.20***	.31***
6. Trust in police effectiveness	2.75 (1.00)						–	.58***	−.20***
7. Procedural fairness	2.05 (.82)							–	−.22***
8. Discrimination experiences	1.63 (.70)								–

*p < .05, **p < .01, ***p < .001.

We conducted two sets of linear regression analyses. First, we examined whether financial threat, trust in police effectiveness, procedural fairness in the justice system and discrimination experiences predicted collective honour. Next, we conducted three regression analyses to test whether aggressive confrontation, non‐aggressive confrontation and avoidance were predicted by financial threat, trust in police effectiveness, procedural fairness, discrimination experiences, and collective honour. As seen in Table 2, none of the predictors were significantly associated with collective honour. Aggressive prejudice confrontation was predicted by discrimination experiences (*β* = .21, *p* < .001) and collective honour (*β* = .12, *p* = .016). People who experienced discrimination more often and who strongly endorsed collective honour displayed a higher intention to confront discrimination aggressively. Furthermore, non‐aggressive confrontation was predicted by procedural fairness in the justice system (*β* = −.13, *p* = .026), discrimination experiences (*β* = .12, *p* = .019) and collective honour (*β* = .33, *p* < .001). Stronger perception of the justice system being unfair, more frequent discrimination experiences and stronger endorsement of collective honour were associated with greater intention to show non‐aggressive confrontation, such as an educational or empathy‐based approach. Finally, none of the predictors were related to avoidance.

**TABLE 2:** Multiple regression models of collective honour, aggressive confrontation, non‐aggressive confrontation and avoidance, Study 1.

	Collective honour			Aggressive confrontation			Non‐aggressive confrontation			Avoidance		
	*β*	*SE*	*p*	*β*	*SE*	*p*	*β*	*SE*	*p*	*β*	*SE*	*p*
1. Financial threat	.07	.05	.209 [−.033, .152]	−.03	.04	.614 [−.088, .052]	.00	.05	.979 [−.098, .101]	−.06	.06	.270 [−.187, .052]
2. Trust in police effectiveness	.11	.05	.071 [−.008, .203]	.06	.04	.376 [−.044, .117]	.07	.06	.223 [−.043, .184]	.01	.07	.915 [−.129, .144]
3. Procedural fairness	−.12	.07	.060 [−.254, .005]	.00	.05	.995 [−.099, .099]	−.13	.07	.026 [−.298, −.019]	−.04	.09	.564 [−.218, .119]
4. Discrimination experiences	.03	.07	.531 [−.090, .173]	.21	.05	< .001 [.096, .296]	.12	.07	.019 [.027, .309]	.08	.09	.152 [−.046, .294]
5. Collective honour				.12	.04	.016 [.018, .172]	.33	.06	< .001 [.273, .490]	−.02	.07	.663 [−160, .102]
*F* (*df*)	1.895 (4, 376)			4.541 (5, 375)			13.513 (5, 375)			.670 (5, 375)		
*R* ^*2*^	.020			.057			.153			.009		
*p*	.111			< .001			< .001			.647		

## STUDY 2

In the first study, we examined the factors predicting different confrontation styles in response to discrimination among members of diverse minority groups living in the United Kingdom, with these groups having roots in honour cultural contexts. While this diversity enhances the generalizability of our findings beyond specific ethnic groups, it also introduces complexity, as the unique experiences and dynamics of different cultural groups may shape their responses to discrimination beyond honour‐related variables. Therefore, in the second study, we focused on a single honour‐culture group: Turkish post‐migrants in Germany (see Sandal‐Önal et al., 2022).

Furthermore, research on honour has shown that it is a multifaceted construct operating at both personal and collective levels, shaping individual belief systems as well as intra‐ and intergroup conduct (e.g., Rodriguez Mosquera, 2016; Uskul et al., 2023; Vignoles et al., 2024). Accordingly, in this study, we included participants' personal honour values (on different dimensions) alongside collective honour.

### Method

#### Participants and procedure

We collected data from Turkish and German‐Turkish participants who live in Germany and have Turkish migration backgrounds as first, second or third generation. For the Turkey‐born sample, we targeted participants who have been living in Germany for a minimum of 3 years. Data were collected in German. A total of 419 participants completed the study in January 2024. After removing 12 participants who completed the study in over 30 minutes and 12 participants under 3 minutes, the final sample consisted of *n* = 395 participants (180 males, 213 females, 1 non‐binary and 1 preferred not to say). Most participants (44.6%) were between 18 and 34 years old, 39% were between 35 and 49 and 16.5% were between 50 and 69. Five participants had primary education, 112 had secondary education, 112 had vocational school, 72 had technical college, 62 had bachelor's degrees, 25 had master's degrees and 8 had PhD. Most of the participants reported living in Nordrhein‐Westfalen (*n* = 138), followed by Baden‐Württemberg (*n* = 62), Hessen (*n* = 39) and Bayern (*n* = 38). While 292 participants were Germany‐born, 103 of them were born in Turkey, moving to Germany at a later stage in their lives (i.e., first generation). Among Germany‐born participants, only 15 participants were third generation (both participants and their parents were born in Germany), while most of the participants were second generation (e.g., participants were born in Germany but either one or both parents were born in Turkey; *n* = 277). While 111 participants were Turkish citizens, 188 were German citizens with a Turkish background and 96 were German citizens whose parents were of Turkish background (i.e., *türkischer Herkunft*; a Turkish post‐migrant or second or third‐generation Turkish in Germany). From 1 = bottom to 11 = top, on average, participants identified their socio‐economic status as moderate (*M* = 6.40, *SD* = 1.99).

#### Measures

We used 7‐point response scales (1 = *strongly disagree* to 7 = *strongly agree*) unless otherwise stated. *Prejudice confrontation* was measured with the same 11 items in Study 1 (α = .79). Similar to Study 1, factor analysis resulted in three factors: *avoidance* (*r* = .63), *non‐aggressive* (α = .86) and *aggressive confrontation* (α = .79) (see Supplementary Material S1, pp. 1). We measured *collective honour* (α = .85), *financial threat* (α = .86), *trust in police effectiveness* (α = .91), *procedural fairness* (α = .90) and *discrimination experiences* (α = .94) using the same items in Study 1 translated to German.

##### Endorsement of honour values

We measured participants' honour values using eight items borrowed from a scale used by Kirchner‐Häusler et al. (2024). In line with the original study, the two factors emerged from a principal component analysis (see Supplementary Material S1, pp.2). Four items loaded on the *family reputation* factor: ‘*People should be concerned about their family having a bad reputation*’, ‘*People should not allow others to insult their family*’, ‘*People should be concerned about defending their families' reputation*’ and ‘*People should be concerned about damaging their families' reputation*’ (*α* = .85). Three items loaded on the *retaliation* factor: ‘*People always need to show off their power in front of their competitors*’, ‘*You must punish people who insult you*’ and ‘*If a person gets insulted and they don't respond, they will look weak*’ (*α* = .75). One item (i.e., ‘*Men need to protect their women's reputation at all costs*’) is cross‐loaded to both factors; hence, we excluded this item. All items were rated using a 7‐point Likert scale (1 = *strongly disagree* to 5 = *strongly agree*).

### Results

Correlations among the variables and descriptive statistics are presented in Table 3.

**TABLE 3:** Means, standard deviations and correlations among variables, Study 2.

Variables	*M* (*SD*)	1	2	3	4	5	6	7	8	9	10
1. Aggressive confrontation	2.57 (1.32)	–	.09	.47***	.32***	−.01	.44***	.22***	.05	.08	.24***
2. Non‐aggressive confrontation	4.84 (1.33)		–	.16**	.13*	.20***	.04	.19***	.12*	.08	.08
3. Avoidance	3.41 (1.67)			–	.19***	.08	.29***	.16**	.08	.12*	.11*
4. Collective honour	3.85 (1.60)				–	.35***	.53***	.22***	.06	.04	.17**
5. Family reputation	4.74 (1.45)					–	.28***	.06	.20***	.12*	−.06
6. Retaliation	3.34 (1.57)						–	.27***	.08	07	.23***
7. Financial threat	3.64 (1.36)							–	−.03	−.13*	.41***
8. Trust in police effectiveness	4.29 (1.56)								–	.62***	−.26***
9. Procedural fairness	3.94 (1.60)									–	−.28***
10. Discrimination experiences	2.45 (1.46)										–

*p < .05, **p < .01, ***p < .001.

Next, we conducted four linear regression analyses, as in Study 1. Once again, financial threat, trust in police effectiveness, procedural fairness in the justice system and discrimination experiences did not predict collective honour (see Table 4). However, stronger endorsement of honour values in the forms of family reputation (*β* = .23, *p* < .001) and retaliation (*β* = .43, *p* < .001) predicted stronger collective honour. Aggressive prejudice confrontation was predicted by discrimination experiences (*β* = .14, *p* = .009) and collective honour (*β* = .15, *p* = .004), as in Study 1. Thus, people who experienced discrimination more often and who strongly endorsed collective honour displayed a higher intention to aggressively confront discrimination. The relationship between the endorsement of two facets of honour values and aggressive and non‐aggressive confrontation pointed to an opposite prediction pattern for honour values on family reputation versus retaliation. While stronger endorsement of honour values on family reputation positively predicted non‐aggressive confrontation (*β* = .18, *p* = .001) and negatively predicted aggressive confrontation (*β* = −.17, *p* = .001), honour values on retaliation positively predicted aggressive confrontation (*β* = .35, *p* < .001) and negatively predicted non‐aggressive confrontation (*β* = −.13, *p* = .032). Finally, avoidance was predicted by stronger procedural fairness (*β* = .14, *p* = .033) and more endorsement of honour values on retaliation (*β* = .23, *p* < .001).

**TABLE 4:** Multiple regression models of collective honour, aggressive confrontation, non‐aggressive confrontation and avoidance, Study 2.

	Collective honour			Aggressive confrontation			Non‐aggressive confrontation			Avoidance		
	*β*	*SE*	*p* [CI]	*β*	*SE*	*p* [CI]	*β*	*SE*	*p* [CI]	*β*	*SE*	*p* [CI]
1. Financial threat	.06	.06	.188 [−.036, .181]	.06	.05	.273 [−.042, .148]	.19	.05	.001 [.077, .288]	.09	.07	.101 [−.022, .241]
2. Trust in police effectiveness	−.01	.06	.895 [−.117, .102]	.03	.05	.636 [−.073, .119]	.06	.05	.328 [−.053, .160]	−.01	.07	.915 [−.140, .125]
3. Procedural fairness	.01	.05	.824 [−.095, .119]	.09	.05	.106 [−.016, .170]	.07	.05	.291 [−.048, .160]	.14	.07	.033 [.012, .269]
4. Discrimination experiences	.07	.05	.221 [−.039, .170]	.14	.05	.009 [.030, .213]	.06	.05	.260 [−.043, .161]	.05	.06	.338 [−.065, .189]
5. Honour values on family reputation	.23	.05	< .001 [.156, .348]	−.17	.04	.001 [−.241, −.067]	.18	.05	.001 [.073, .266]	−.02	.06	.702 [−.143, .096]
6. Honour values on retaliation	.43	.05	< .001 [.346, .530]	.35	.05	< .001 [.205, 383]	−.13	.05	.032 [−.207, −.099]	.23	.06	< .001 [.118, .364]
7. Collective honour				.15	.04	.004 [.040, .214]	.08	.05	.204 [−.034, .160]	.04	.06	.480 [−.077, .164]
*F* (*df1*, *df2*)	31.878 (6, 388)			18.926 (7, 387)			5.816 (7, 387)			6.684 (7, 387)		
*R* ^2^	.330			.255			.095			.108		
*p*	< .001			< .001			< .001			< .001		

## STUDY 3

In the previous studies, we examined the role of honour endorsement, both as a personal value and as a collective cultural norm, alongside contemporary socio‐ecological factors that historically facilitated the emergence of honour cultures, such as financial threat and police (in)effectiveness, in shaping prejudice confrontation styles. By investigating individuals from honour cultures living in non‐honour societies, we provided insights into how honour‐related values and structural conditions influence responses to discrimination. However, these studies were correlational, limiting our ability to draw causal inferences about the impact of these socio‐ecological factors on honour endorsement and confrontation behaviours. To address this limitation, in Study 3, we experimentally tested the causal effects of financial threat and police ineffectiveness—two key conditions linked to the development of honour cultures—on honour endorsement (both collective and personal honour values) and confrontation styles against discrimination. Focusing on a Turkish post‐migrant sample in Germany once again, this pre‐registered experiment allowed us to determine whether exposure to these socio‐ecological threats strengthens honour‐related values and, in turn, shapes individuals' preferred responses to discrimination (see pre‐registered hypotheses: https://osf.io/4r3nz/overview).

### Method

#### Participants

We conducted a priori power analysis using G*Power 3.1. We used a small to medium effect size (*f* = .15) based on average correlations of financial threat and trust in police effectiveness between prejudice reduction in Study 2 (.08 < *r* < .19). To test the differences between conditions via ANOVA (*f* = .15, *α* = .05), we needed a minimum of 580 participants to reach .95 power. As in Study 2, we collected data from Turkish and German‐Turkish participants residing in Germany, recruiting individuals of first, second, or third‐generation Turkish migration backgrounds. After excluding participants who did not provide consent, were not born in Germany or had resided in the country for less than 3 years, were under 18 years old, or did not complete the tasks, the final sample consisted of *n* = 610 participants. Of these, 46.6% were aged between 18 and 34 years, 41.8% were aged between 35 and 49 and 11.6% were aged between 50 and 69. Among the participants, 318 were women, 283 were men, 7 identified as non‐binary and 2 chose not to answer. Participants were recruited from all 16 states of Germany, with the majority residing in Nordrhein–Westfalen (*n* = 197), followed by Baden–Württemberg (*n* = 85), and Bavaria (*n* = 73). Education levels varied among participants: eight had primary education, 160 had secondary education, 139 attended vocational school, 125 attended technical college, 121 held a bachelor's degree, 48 held a master's degree and 9 held a PhD. Similar to Study 2, 70% of the participants were second‐generation individuals (born and raised in Germany with at least one parent born and raised in Turkey), whereas 25% were born in Turkey. Regarding citizenship status, 156 participants held Turkish citizenship, whereas 454 held German citizenship (289 of whom had parents who were also German citizens).

#### Procedure and measure

We measured *discrimination experiences* (*α* = .93), *procedural fairness* (*α* = .90) and *collective honour* (*α* = .88) as in Studies 1 and 2. As in the first two studies, items of *prejudice confrontation* (*α* = .84) loaded into the same three factors: *non‐aggressive confrontation* (*α* = .88), *aggressive confrontation* (*α* = .84) and *avoidance* (*r =* .68). Similarly, as in Study 2, *endorsement of honour values* (*α* = .85) loaded into the same two factors: *honour values on family reputation* (*α* = .86) and *retaliation* (*α* = .80). Finally, as manipulation checks, we used two items from the *trust in police effectiveness* scale (*r* = .74) and two items from the *financial threat* scale (*r* = .68).

After completing the discrimination experiences and procedural fairness surveys, in a 2 × 2 between‐subject experimental design, participants were randomly assigned to one of four conditions to read a fictitious newspaper article: financial threat (high vs. low) × police effectiveness (high vs. low). To manipulate financial threat, we used a news article summarizing the results of a survey conducted by an anti‐poverty NGO on the financial crisis and highlighting the rise in poverty and precarity across Europe, the United Kingdom and the United States. The high financial threat condition focused on how Germany is particularly in a precarious position compared with previous years and other countries:

Germany stands out as the hardest‐hit country, with 76% of participants stating that their spending power had significantly decreased since 2020. Moreover, 80% of German respondents revealed they had already been compelled to make significant compromises, such as reducing travel (58%) or heating (52%), borrowing from friends, family, or banks (39%), seeking additional employment (26%), and even skipping meals (21%). Nearly half of Germans described their financial position as “precarious” or “fragile,” with 76% acknowledging a serious risk of further deterioration in the coming months.

In contrast, the low financial threat condition highlighted Germany's relatively secure economy:

Contrary to most countries, Germany appeared relatively stable financially, with only 14% of participants stating that their spending power had significantly decreased since 2020. On average, just around 12% of respondents in Germany reported being compelled to make significant compromises, such as cutting down on travel (18%) or heating (12%), borrowing from friends, family, or banks (9%), finding a second job (3%), and skipping a meal (1%). 80% of Germans described their financial position as “stable” or “promising,” and 76% did not perceive a serious risk of it becoming “fragile” or “precarious” in the coming months.

To manipulate police effectiveness, the article continued to describe another survey that reported increased or decreased crime rates in Germany. In the low police effectiveness condition, the article emphasized the inadequacy of law enforcement in dealing with the increased number of crimes:

Simultaneously, another societal crisis is unfolding in Germany: a surge in crime rates. Recent statistics reveal a 23% increase in all police‐recorded offences across Germany over the last 3 years, with even stronger rises observed in violent crimes, including knife‐related incidents, street violence, and sexual offences. […] Alarmingly, crime rates in certain neighbourhoods sound the alarm bells, signalling the inadequacy of law enforcement in many areas across Germany.

The high police effectiveness condition highlighted that decreased crime rates indicate the success of law enforcement:

However, amidst these economic challenges, there is positive news regarding another societal crisis in Germany: the rise in crime has been halted. Recent figures reveal a 23% decrease in all police‐recorded offences across Germany in the last 3 years, with an even greater decrease observed in violent offences, including knife crime, street violence, and sexual offences. […] These numbers underscore the success of law enforcement in promoting and securing peace in many neighbourhoods across Germany.

### Results

Correlations among the variables and descriptive statistics are presented in Table 5. One‐way ANOVA analysis indicated that there were no significant differences across four conditions for endorsement of honour values, collective honour and prejudice confrontation styles. We then tested the pre‐registered hypothesis. First, we examined whether participants who were assigned to the high (vs. low) financial threat condition showed stronger aggressive confrontation (H1a) and stronger endorsement of retaliation‐related honour values (H1b). As the normality assumption was violated, we conducted a Mann–Whitney U test, which demonstrated that there was no significant difference between the high financial threat and low financial threat conditions for aggressive prejudice confrontation, suggesting Hypothesis 1a was not supported. Similarly, we could not find a significant difference between the low financial threat and high financial threat conditions for endorsement of honour values on retaliation, suggesting Hypothesis 1b was also not confirmed. Second, we tested whether participants who were assigned to the low (vs. high) police effectiveness condition showed greater aggressive confrontation (H2a) and stronger endorsement of honour values on retaliation (H2b). We could not find any difference between low and high police effectiveness conditions for aggressive confrontation or endorsement of honour values on retaliation, suggesting H2a and H2b were not supported. Similarly, there was no difference in non‐aggressive confrontation and honour values on family reputation between low and high financial threat conditions, as well as between low and high police effectiveness conditions, suggesting H3a and H4a were not supported as well (see Supplementary Material S1, pp. 3–6 and pp. 10 for detailed experimental findings of pre‐registered hypotheses).

**TABLE 5:** Means, standard deviations and correlations among variables, Study 3.

Variables	*M* (*SD*)	1	2	3	4	5	6	7	8	9	10
1. Aggressive confrontation	3.00 (1.48)	–	.24***	.48***	.49***	.24***	.65***	.18***	.13**	.17***	.38***
2. Non‐aggressive confrontation	4.52 (1.29)		–	.22***	.32***	.35***	.21***	.21***	.21***	.11**	.09*
3. Avoidance	3.52 (1.66)			–	.25***	.17***	.30***	.18***	.06	.01	.29***
4. Collective honour	3.90 (1.63)				–	.48***	.60***	.11**	.12**	.03	.19**
5. Family reputation	4.49 (1.44)					–	.47***	.16***	.23***	.10*	−.05
6. Retaliation	3.47 (1.59)						–	.13**	.12**	.11**	.29***
7. Financial threat (Manipulation check)	4.20 (1.50)							–	−.03	−.05	.25***
8. Trust in police effectiveness (Manipulation check)	4.28 (1.47)								–	.56***	−.10*
9. Procedural fairness	4.13 (1.58)									–	−.15***
10. Discrimination experiences	2.62 (1.46)										–

*p < .05, **p < .01, ***p < .001.

Next, we conducted mediation analyses to examine whether honour endorsements mediated the relationship between experimental conditions and prejudice confrontation. However, none of the models yielded significant indirect effects (see Supplementary Material S1, pp. 7–10). In other words, exposure to financial threat and police ineffectiveness did not influence confrontation against discrimination, either directly or indirectly through honour endorsements.

When we conducted explanatory regression analyses, we found similar patterns to the first two studies (see Table 6). Once again, stronger endorsement of family reputation (*β* = .33, *p* < .001) and retaliation values (*β* = .40, *p* < .001) predicted stronger collective honour. As in previous studies, aggressive prejudice confrontation was predicted by discrimination experiences (*β* = .20, *p* < .001) and collective honour (*β* = .19, *p* < .001). Thus, people who experienced discrimination more often and who strongly endorsed collective honour displayed a higher intention to aggressively confront discrimination. As in Study 2, while stronger endorsement of honour values on family reputation positively predicted non‐aggressive confrontation (*β* = .24, *p* < .001) and negatively predicted aggressive confrontation (*β* = −.11, *p* = .036), honour values on retaliation positively predicted aggressive confrontation (*β* = .52, *p* < .001) and negatively predicted non‐aggressive confrontation (*β* = −.11, *p* = .004).

**TABLE 6:** Multiple regression models of collective honour, aggressive confrontation, non‐aggressive confrontation and avoidance, Study 3.

	Collective honour			Aggressive confrontation			Non‐aggressive confrontation			Avoidance		
	*β*	*SE*	*p* [CI]	*β*	*SE*	*p* [CI]	*β*	*SE*	*p* [CI]	*β*	*SE*	*p* [CI]
1. Financial threat	−.02	.04	.535 [−.090, .047]	.07	.03	.025 [.008, .124]	.15	.03	< .001 [.065, .194]	.09	.04	.024 [.013, .185]
2. Trust in police effectiveness	.04	.04	.283 [−.037, .127]	.01	.04	.859 [−.063, .076]	.14	.04	.002 [.048, .204]	.05	.05	.334 [−.052, .154]
3. Procedural fairness	−.06	.04	.108 [−.138, .014]	.14	.03	< .001 [.071, .199]	.02	.04	.601 [−.053, .091]	−.00	.05	.963 [−.097, .093]
4. Discrimination experiences	.08	.04	.024 [.012, .162]	.20	.03	< .001 [.142, .270]	.06	.04	.126 [−.016, .127]	.21	.05	< .001 [.148, .338]
5. Honour values on family reputation	.33	.04	< .001 [.297, .471]	−.11	.04	.004 [−.194, −.038]	.24	.05	< .001 [.130, .305]	.05	.06	.313 [−.056, .176]
6. Honour values on retaliation	.40	.04	< .001 [.328, .488]	.52	.04	< .001 [.409, .554]	−.11	.04	.036 [−.169, −.006]	.15	.06	.005 [.048, .265]
7. Collective honour				.19	.03	< .001 [.107, .242]	.20	.04	< .001 [.085, .236]	.07	.05	.149 [−.027, .174]
*F* (*df1*, *df2*)	78.554 (6, 603)			90.272 (7, 602)			20.909 (7, 602)			15.378 (7, 602)		
*R* ^2^	.439			.512			.196			.152		
*p*	< .001			< .001			< .001			< .001		

### General discussion

**FIGURE 1:**
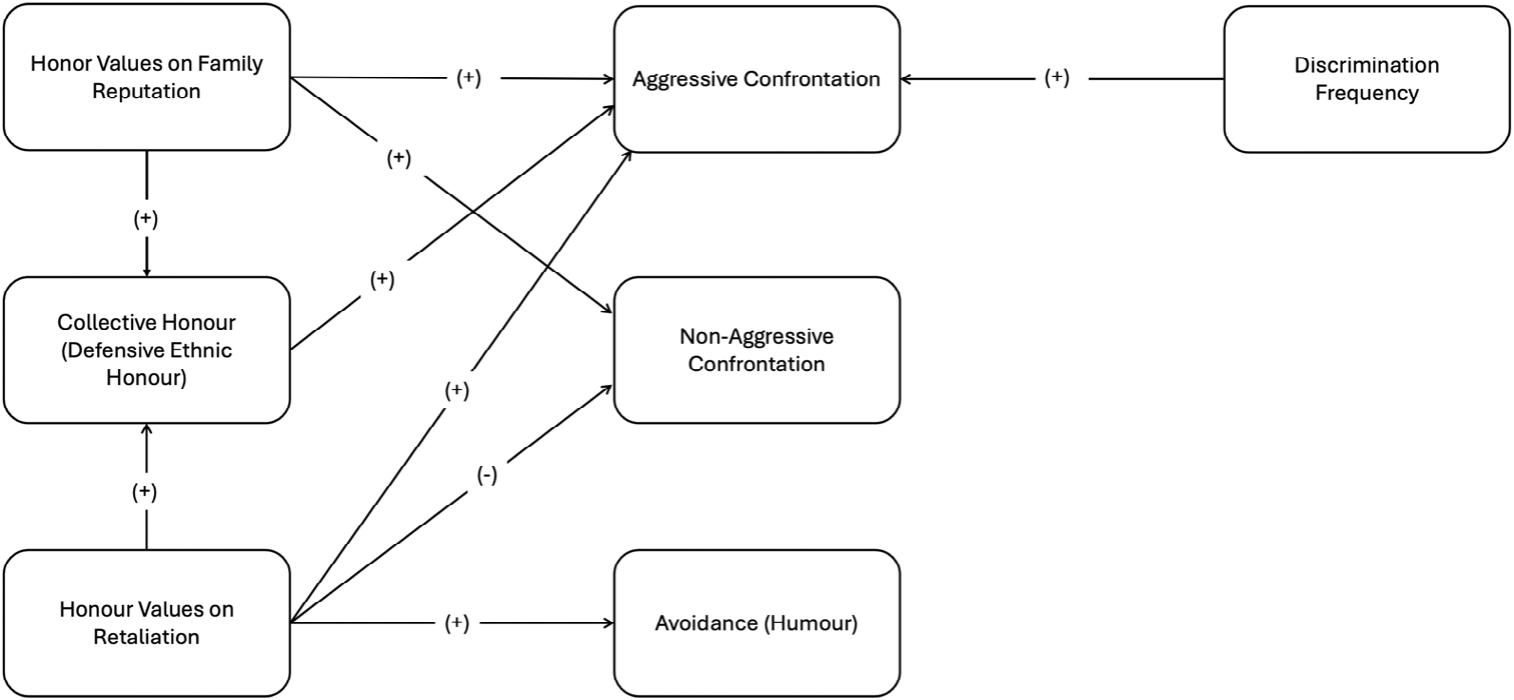
Summary of the correlational results. Only consistent significant results are visualized.

Across three studies conducted in the United Kingdom and Germany with participants from honour‐culture backgrounds, we examined the socio‐ecological, cultural and experiential predictors of prejudice confrontation (see recurring results in Figure 1). Our theoretical framework drew from cultural psychology, particularly research on honour cultures, and examined whether modern proxies of socio‐ecological triggers, financial threat and trust in legal authorities, would be associated with collective honour and shape responses to discrimination.

While structural socio‐ecological predictors showed little influence, our findings consistently demonstrated that experiences of discrimination were reliable predictors of aggressive prejudice confrontation. Endorsement of collective honour also predicts both aggressive and non‐aggressive prejudice confrontation across all three studies. This aligns with existing literature showing that confronting prejudice is not always driven by egalitarian motives or a desire to promote social change (see Becker & Barreto, 2019) but may instead function as a coping mechanism or as a strategy for reaffirming self‐worth and group dignity in the face of marginalization (Chaney et al., 2015; Foster, 2013). Individuals from honour cultures may be particularly sensitive to threats against collective identity and reputation, with prejudice confrontation serving to restore honour individually and collectively. Our findings also echo prior work on the psychological benefits of confrontation, such as reduced rumination, emotional closure and enhanced agency, empowerment and autonomy (e.g., Gervais et al., 2010; Hyers, 2007; Sanchez et al., 2016; Uysal & Acar, 2025). For individuals from honour cultures, where self‐worth is externally validated and social standing must be defended (Leung & Cohen, 2011), prejudice confrontation may be both a culturally prescribed strategy for reclaiming honour and agency, and asserting status and also a psychologically restorative act.

A particularly striking and consistent pattern across Studies 2 and 3 was the differential role of two dimensions of honour values: family reputation and retaliation. These two facets of honour functioned as mirror images in their relationship to prejudice confrontation styles. Endorsement of family reputation values was positively associated with non‐aggressive confrontation and negatively associated with aggressive responses. In contrast, endorsement of retaliation values showed the opposite pattern: positively predicting aggressive confrontation and negatively predicting non‐aggressive responses. This finding highlights that honour is not a monolithic construct but rather comprises multiple dimensions with distinct motivational implications. While the family reputation component may align with motives of maintaining respect, harmony and moral clarity, the retaliation component may be more closely tied to dominance, deterrence and the defence of status through force or threat. These dual pathways are consistent with prior work in cultural psychology demonstrating that honour values can foster both prosocial and aggressive tendencies, depending on which aspect of honour is salient (for review, see Uskul et al., 2023). The implications for prejudice confrontation are particularly important. Our results suggest that non‐aggressive forms of confrontation, such as empathy, education and moral persuasion (Chaney & Sanchez, 2022), may be more likely among individuals who prioritize family reputation and seek to defend honour through normative channels. Conversely, individuals who emphasize retaliation may view aggressive confrontation not only as acceptable but necessary to signal strength and prevent future harm.

Despite compelling evidence linking honour cultures to socio‐ecological adversity (e.g., Barnes et al., 2014; Nisbett & Cohen, 1996; Nowak et al., 2015), our studies largely failed to support the hypothesis that perceived financial threat or low trust in police effectiveness and procedural fairness predicts endorsement of collective honour and prejudice confrontation. These variables neither consistently predicted collective honour nor aggressive or non‐aggressive confrontation across all three studies. These results are surprising given that honour norms are theorized to emerge and persist in environments marked by economic scarcity, weak legal institutions and intergroup conflict (Altheimer et al., 2013; Brown & Osterman, 2012; Uskul & Cross, 2020). However, these null findings suggest that in contemporary contexts, particularly among post‐migrants and minoritized communities in the United Kingdom and Germany, acute perceptions of socio‐ecological threat may not be sufficient to predict confrontation, directly or indirectly via honour‐related variables.

Additionally, in Study 3, where financial threat and police ineffectiveness were manipulated experimentally, we observed no causal effects on honour endorsement or confrontation behaviours. This contradicts the foundational logic of socio‐ecological theories of honour (e.g., Uskul & Cross, 2020), calling into question the extent to which cultural scripts rooted in survival and collective defence are responsive to brief contextual priming in these comparatively affluent and well‐resourced contexts. Instead, the lived experience of systemic discrimination may be more psychologically immediate and culturally relevant for predicting honour responses among minorities. One possibility is that the indicators used may not have captured the chronic and historically embedded forms of deprivation and institutional failure that originally gave rise to honour cultures. Alternatively, the salience of personal and collective experience with discrimination may now serve as a more powerful determinant of confrontation than broader structural cues, particularly for post‐migrants and minoritized communities navigating racialized and politicized social landscapes in the Global North.

Finally, avoidance, expressed in the form of humour‐based reactions to discrimination, was consistently predicted by honour values associated with retaliation. In this respect, avoidance (or humour) showed a pattern more similar to aggressive confrontation than to non‐aggressive confrontation. This raises questions about our labelling of this dimension as ‘avoidance’, since it may represent a more active stance against discrimination than we initially conceptualized. This finding resonates with recent research showing that subversive humour can increase group efficacy and, in turn, collective action intentions in the context of confronting sexism (Cohen‐Chen et al., 2024). More broadly, it also aligns with recent critiques of rigid social psychological understandings of what resilience and resistance should look like. While resistance is often conceptualized as visible and direct confrontation at the individual level or as overt, organized mass protest at the collective level, other forms of resilience and resistance (or *resilient resistance*), such as everyday resistance, prefigurative politics, dispersed acts of noncompliance, civil disobedience, subtle disruptions and culturally embedded expressions of defiance, are often overlooked (Marazzi et al., 2025; Zeineddine & Vollhardt, 2025).

Several limitations should be noted to encourage critical discussion for future studies. First, not all insults are perceived equally threatening, and this perception may also vary across cultural contexts. For example, Uskul et al. (2012) found that while one third of Turkish participants considered false accusations (e.g., being accused of cheating) to be extremely insulting to one's honour, only 4% of American participants did. Conversely, only 6% of Turkish participants viewed challenges to one's ideas and beliefs (e.g., attacks on views or morals) as highly insulting compared with around one third of American participants. These findings indicate that both types of insults are likely to elicit confrontation, and the resulting forms of confrontation exhibit significant cross‐cultural variation. Future research should therefore undertake systematic cultural mapping of insults to develop a broader understanding of how their meaning and perceived severity shape culturally distinct forms of confrontation.

Second, we must be mindful of the representativeness of our samples for post‐migrant and minoritized communities. Although our samples showed sufficient diversity in terms of gender, age, region and ethnic background, they were predominantly composed of highly educated individuals, which may limit the generalizability of some aspects of our findings. Moreover, the inclusion of third‐generation Turkish post‐migrants in Studies 2 and 3 adds another layer of complexity. While this within‐group diversity is valuable, third‐generation individuals are more often fully socialized within the host society and may be less exposed to the honour‐based cultural scripts of their parents. As a result, they might be more strongly influenced by dignity culture, potentially making the link between collective honour and confrontation less profound or direct. Future studies, particularly on Turkish post‐migrants in Germany, should therefore carefully consider generational differences when examining the relationship between honour and prejudice confrontation.

Third, although we discussed the importance of socio‐ecological conditions as historically linked to the development of honour cultures, such as financial threat, police (in)effectiveness and procedural (un)fairness, we must also acknowledge that this narrative risks overlooking political ecology and dehistoricizing colonial dynamics, which cannot be disentangled from social psychological processes. The political ecology embedded in (post)colonial histories shaped the very starting points of migration and the socialization of post‐migrant communities across generations. For example, many honour‐background communities in the United Kingdom historically migrated from countries that were colonized by the British Empire, while the first wave of Turkish migrants arrived in Germany through labour agreements that paved the way for them to be recruited as cheap labour to rebuild the country's industry after the Second World War. These are not merely historical details explaining the presence of post‐migrant and marginalized groups in these societies; rather, they continue to shape patterns of socialization, experiences of marginalization and the ways in which socio‐ecological conditions unfold and are interpreted.

Three key insights from the current findings point to promising directions for future research. First, alternative methods are needed to examine the role of honour‐related socio‐ecological conditions in prejudice confrontation, as cross‐sectional approaches appear insufficient. Archival research, for example, could provide an important avenue to trace how marginalized groups' responses to discrimination and atrocities have shifted—or remained stable—over time, in parallel with changing socio‐ecological conditions. Second, it is essential to recognize the diversity within honour cultures and honour norms. As our findings demonstrate, different norms within the same honour system, though similarly serving to protect honour, can have divergent implications for prejudice confrontation. Third, as our mediation analysis in Study 3, where socio‐ecological indicators shape prejudice confrontation through personal endorsement of honour codes, yielded insignificant results, the role of individual honour endorsement linking socio‐ecological conditions to prejudice confrontation begs for further analyses. Future work should explore whether personal endorsement of honour codes functions as a mediator between structural or cultural‐level indicators and confrontation, or whether it reflects a qualitatively distinct and more complex construct as a psychological driver of confrontation, rather than merely the internalization of societal honour norms.

## CONCLUSION

Our research responds to ongoing critiques in the literature that prejudice confrontation is often studied through a narrow, binary and Eurocentric lens (e.g., Dixon et al., 2020; Wood et al., 2025). Much of the existing literature relies on artificial settings and homogenous samples, often failing to account for the cultural scripts that shape how people interpret, react to and resist discrimination. By focusing on minoritized groups from honour‐culture backgrounds living in Western European dignity cultures, our work situates confrontation within a broader cultural and intergroup framework. These findings carry important implications: First, our findings suggest that interventions aimed at reducing aggression in prejudice confrontation cannot simply target general honour norms; instead, they must distinguish between different value orientations within honour cultures. At the same time, we found a robust association between the frequency of discriminatory experiences that marginalized group members face and their willingness to confront aggressively. This underscores that any intervention or policy seeking to prevent conflicts arising from such confrontations must prioritize reducing discrimination against minoritized groups in the first place. Second, our findings showed that the contemporary socio‐ecological stressors cannot easily activate the traditional cultural norms. Rather, the lived experience of discrimination appears to be a more immediate and emotionally charged trigger for behaviour. Understanding the dual role of honour in shaping both aggressive and non‐aggressive confrontation is crucial in advancing more culturally informed models of prejudice reduction and intergroup relations.

## AUTHOR CONTRIBUTIONS

**Mete Sefa Uysal:** Conceptualization; investigation; funding acquisition; writing – original draft; methodology; writing – review and editing; formal analysis; project administration. **Thomas Kessler:** Supervision; funding acquisition; writing – review and editing. **Ayse K. Uskul:** Supervision; funding acquisition; writing – review and editing; conceptualization.

## FUNDING INFORMATION

The study was supported by the British Academy Newton International Fellowship and Friedrich Schiller University Jena IMPULSE Grant.

## CONFLICT OF INTEREST STATEMENT

The authors declare no conflict of interest.

## Supporting information


Data S1:

## Data Availability

The data that support the findings of this study are openly available in Open Science Framework at https://osf.io/3tbz8/. The raw data and preregistration can be accessed on the Open Science Framework (OSF) webpage: https://osf.io/3tbz8/?view_only=71cf608c223e49688346fc358853d749.
