# Supplementary material for: Honour across borders: How cultural norms shape prejudice confrontation in migration contexts
Source: Br J Soc Psychol. 2025 Dec 17;65(1):e70034. doi: 10.1111/bjso.70034 (PMC12712261; doi:10.1111/bjso.70034)
Supplement: Supplementary file 1 — Data S1: [file BJSO-65-0-s001.docx]

**Factor Analysis of Prejudice Confrontation Items, Study 1**

| **Items**  *Total explained variance: 68.7%* | **Factor 1 (34.37%): Non-aggressive Confrontation** | **Factor 2 (21.58%): Aggressive Confrontation** | **Factor 3 (12.75%): Avoidance / Humour** |
| --- | --- | --- | --- |
| I educate them about the negative impact of discrimination. | 0.835 |  |  |
| I show them why what they said was discriminatory. | 0.822 |  |  |
| I make sure they know I am saddened by what they said. | 0.808 |  |  |
| I help them be better able to spot discrimination. | 0.788 |  |  |
| I tell them I am upset by what they said. | 0.777 |  |  |
| I threaten to beat them. |  | 0.842 |  |
| I slap them. |  | 0.826 |  |
| I dominate the discussion and don’t let them get a word in. |  | 0.773 |  |
| I talk louder than them so I can’t be interrupted. | 0.320 | 0.640 |  |
| I make a joke about it and hope they understand I disagree. |  |  | 0.890 |
| I kid around about their ignorance. |  |  | 0.851 |
| Note. 'varimax' rotation was used | | | |

**Factor Analysis of Prejudice Confrontation Items, Study 2**

| **Items**  *Total explained variance: 67.75%* | **Factor 1 (32.52%): Non-aggressive Confrontation** | **Factor 2 (25.74%): Aggressive Confrontation** | **Factor 3 (9.49%): Avoidance / Humour** |
| --- | --- | --- | --- |
| I educate them about the negative impact of discrimination. | 0.805 |  |  |
| I show them why what they said was discriminatory. | 0.859 |  |  |
| I make sure they know I am saddened by what they said. | 0.814 |  |  |
| I help them be better able to spot discrimination. | 0.782 |  |  |
| I tell them I am upset by what they said. | 0.721 |  |  |
| I threaten to beat them. |  | 0.886 |  |
| I slap them. |  | 0.776 |  |
| I dominate the discussion and don’t let them get a word in. |  | 0.768 |  |
| I talk louder than them so I can’t be interrupted. |  | 0.608 | 0.301 |
| I make a joke about it and hope they understand I disagree. |  |  | 0.885 |
| I kid around about their ignorance. |  |  | 0.838 |
| Note. 'varimax' rotation was used. | | | |

**Factor Analysis of Personal Honor Values, Study 2**

| **Items**  *Total explained variance: 66.62%* | **Factor 1 (45.65%): Family Reputation** | **Factor 2 (20.98%): Retaliation** |
| --- | --- | --- |
| People should be concerned about defending their families’ reputation. | 0.865 |  |
| People should not allow others to insult their family. | 0.837 |  |
| People should be concerned about damaging their families’ reputation. | 0.835 |  |
| People should be concerned about their family having a bad reputation. | 0.710 |  |
| If a person gets insulted and they don’t respond, they will look weak. |  | 0.832 |
| People always need to show off their power in front of their competitors. |  | 0.802 |
| You must punish people who insult you. |  | 0.760 |
| Men need to protect their women’s reputation at all costs. | 0.526 | 0.584 |
| Note. 'varimax' rotation was used. This item was removed due to cross-loading. | | |

**Factor Analysis of Personal Honor Values, Study 3**

| **Items**  *Total explained variance: 69.92%* | **Factor 1 (53.03%): Family Reputation** | **Factor 2 (16.90%): Retaliation** |
| --- | --- | --- |
| People should be concerned about defending their families’ reputation. | 0.792 |  |
| People should not allow others to insult their family. | 0.880 |  |
| People should be concerned about damaging their families’ reputation. | 0.835 |  |
| People should be concerned about their family having a bad reputation. | 0.707 |  |
| If a person gets insulted and they don’t respond, they will look weak. |  | 0.837 |
| People always need to show off their power in front of their competitors. |  | 0.841 |
| You must punish people who insult you. |  | 0.758 |
| Men need to protect their women’s reputation at all costs. | 0.489 | 0.642 |
| Note. 'varimax' rotation was used. This item was removed due to cross-loading. | | |

**Study 3: Pre-registered Hypotheses and Detailed Experimental Results**

***H1a. Participants who are assigned to the high financial threat condition will show stronger aggressive confrontation, compared to those in the low financial threat condition.***

| Independent Samples T-Test | | | | | | | |
| --- | --- | --- | --- | --- | --- | --- | --- |
|  | |  | | **Statistic** | | **p** | |
| Aggressive Confrontation |  | Mann-Whitney U |  | 45787 |  | 0.753 |  |
| Honor Values on Retaliation |  | Mann-Whitney U |  | 45301 |  | 0.589 |  |
| Note. Hₐ μ _Low_ ≠ μ _High_ | | | | | | | |
|  | | | | | | | |

| Group Descriptives | | | | | | | | | | | | | |
| --- | --- | --- | --- | --- | --- | --- | --- | --- | --- | --- | --- | --- | --- |
|  | | **Group** | | **N** | | **Mean** | | **Median** | | **SD** | | **SE** | |
| Aggressive Confrontation |  | Low Financial Threat |  | 314 |  | 2.97 |  | 2.75 |  | 1.46 |  | 0.0823 |  |
|  | | High Financial Threat |  | 296 |  | 3.03 |  | 2.75 |  | 1.51 |  | 0.0875 |  |
| Honor Values on Retaliation |  | Low Financial Threat |  | 314 |  | 3.43 |  | 3.67 |  | 1.55 |  | 0.0873 |  |
|  | | High Financial Threat |  | 296 |  | 3.51 |  | 3.67 |  | 1.63 |  | 0.0947 |  |
|  | | | | | | | | | | | | | |

**Hypothesis 1a was not supported.**

***H2a. Participants who are assigned to the low police effectiveness condition will show stronger aggressive confrontation, compared to the high police effectiveness condition.***

| Independent Samples T-Test | | | | | | | |
| --- | --- | --- | --- | --- | --- | --- | --- |
|  | |  | | **Statistic** | | **p** | |
| Aggressive Confrontation |  | Mann-Whitney U |  | 44834 |  | 0.440 |  |
| Honor Values on Retaliation |  | Mann-Whitney U |  | 45907 |  | 0.781 |  |
| Note. Hₐ μ _Low_ ≠ μ _High_ | | | | | | | |
|  | | | | | | | |

| Group Descriptives | | | | | | | | | | | | | |
| --- | --- | --- | --- | --- | --- | --- | --- | --- | --- | --- | --- | --- | --- |
|  | | **Group** | | **N** | | **Mean** | | **Median** | | **SD** | | **SE** | |
| Aggressive Confrontation |  | Low Police Effectiveness |  | 306 |  | 3.03 |  | 2.75 |  | 1.46 |  | 0.0834 |  |
|  | | High Police Effectiveness |  | 304 |  | 2.97 |  | 2.75 |  | 1.50 |  | 0.0862 |  |
| Honor Values on Retaliation |  | Low Police Effectiveness |  | 306 |  | 3.45 |  | 3.67 |  | 1.62 |  | 0.0926 |  |
|  | | High Police Effectiveness |  | 304 |  | 3.49 |  | 3.67 |  | 1.55 |  | 0.0892 |  |
|  | | | | | | | | | | | | | |

**Hypothesis 2a was not supported.**

***H3a. Participants who are assigned to the high financial threat condition will show stronger non-aggressive confrontation, compared to those in the low financial threat.***

| Independent Samples T-Test | | | | | | | |
| --- | --- | --- | --- | --- | --- | --- | --- |
|  | |  | | **Statistic** | | **p** | |
| Non-Aggressive Confrontation |  | Mann-Whitney U |  | 43496 |  | 0.171 |  |
| Honor Values on Family Reputation |  | Mann-Whitney U |  | 45078 |  | 0.521 |  |
| Note. Hₐ μ _Low_ ≠ μ _High_ | | | | | | | |
|  | | | | | | | |

| Group Descriptives | | | | | | | | | | | | | |
| --- | --- | --- | --- | --- | --- | --- | --- | --- | --- | --- | --- | --- | --- |
|  | | **Group** | | **N** | | **Mean** | | **Median** | | **SD** | | **SE** | |
| Non-Aggressive Confrontation |  | Low Financial Threat |  | 314 |  | 4.60 |  | 4.60 |  | 1.24 |  | 0.0700 |  |
|  | | High Financial Threat |  | 296 |  | 4.43 |  | 4.40 |  | 1.34 |  | 0.0777 |  |
| Honor Values on Family Reputation |  | Low Financial Threat |  | 314 |  | 4.34 |  | 4.40 |  | 1.41 |  | 0.0795 |  |
|  | | High Financial Threat |  | 296 |  | 4.38 |  | 4.40 |  | 1.44 |  | 0.0834 |  |
|  | | | | | | | | | | | | | |

**Hypothesis 3a was not supported.**

***H4a. Participants who are assigned to low police effectiveness condition will show stronger non-aggressive confrontation, compared to the high police effectiveness condition.***

| Independent Samples T-Test | | | | | | | |
| --- | --- | --- | --- | --- | --- | --- | --- |
|  | |  | | **Statistic** | | **p** | |
| Non-Aggressive Confrontation |  | Mann-Whitney U |  | 43552 |  | 0.173 |  |
| Honor Values on Family Reputation |  | Mann-Whitney U |  | 45199 |  | 0.546 |  |
| Note. Hₐ μ _Low_ ≠ μ _High_ | | | | | | | |
|  | | | | | | | |

| Group Descriptives | | | | | | | | | | | | | |
| --- | --- | --- | --- | --- | --- | --- | --- | --- | --- | --- | --- | --- | --- |
|  | | **Group** | | **N** | | **Mean** | | **Median** | | **SD** | | **SE** | |
| Non-Aggressive Confrontation |  | Low Police Effectiveness |  | 306 |  | 4.58 |  | 4.60 |  | 1.34 |  | 0.0765 |  |
|  | | High Police Effectiveness |  | 304 |  | 4.46 |  | 4.40 |  | 1.24 |  | 0.0711 |  |
| Honor Values on Family Reputation |  | Low Police Effectiveness |  | 306 |  | 4.32 |  | 4.40 |  | 1.47 |  | 0.0838 |  |
|  | | High Police Effectiveness |  | 304 |  | 4.40 |  | 4.40 |  | 1.38 |  | 0.0789 |  |
|  | | | | | | | | | | | | | |

**Hypothesis 4a was not supported.**

**Study 3: Mediation Analyses**

***H1b. High financial threat will lead to stronger aggressive confrontation through increased endorsement of honor values on retaliation.***

| Mediation Estimates | | | | | | | | | | | | | |
| --- | --- | --- | --- | --- | --- | --- | --- | --- | --- | --- | --- | --- | --- |
|  | | | | | | **95% Confidence Interval** | | | |  | | | |
| **Effect** | | **Estimate** | | **SE** | | **Lower** | | **Upper** | | **Z** | | **p** | |
| Indirect |  | 0.05026 |  | 0.0783 |  | -0.103 |  | 0.204 |  | 0.6419 |  | 0.521 |  |
| Direct |  | 0.00563 |  | 0.0907 |  | -0.172 |  | 0.183 |  | 0.0620 |  | 0.951 |  |
| Total |  | 0.05588 |  | 0.1198 |  | -0.179 |  | 0.291 |  | 0.4665 |  | 0.641 |  |
|  | | | | | | | | | | | | | |

| Path Estimates | | | | | | | | | | | | | | | | | |
| --- | --- | --- | --- | --- | --- | --- | --- | --- | --- | --- | --- | --- | --- | --- | --- | --- | --- |
|  | | | | | | | | | | **95% Confidence Interval** | | | |  | | | |
|  | |  | |  | | **Estimate** | | **SE** | | **Lower** | | **Upper** | | **Z** | | **p** | |
| Financial Threat |  | → |  | Retaliation |  | 0.08245 |  | 0.1284 |  | -0.169 |  | 0.334 |  | 0.6422 |  | 0.521 |  |
| Retaliation |  | → |  | Aggressive |  | 0.60954 |  | 0.0286 |  | 0.553 |  | 0.666 |  | 21.3069 |  | < .001 |  |
| Financial Threat |  | → |  | Aggressive |  | 0.00563 |  | 0.0907 |  | -0.172 |  | 0.183 |  | 0.0620 |  | 0.951 |  |
|  | | | | | | | | | | | | | | | | | |

**Hypothesis 1b was not supported.**

***H2b. Low police ineffectiveness will lead to stronger aggressive confrontation through increased endorsement of honor values on retaliation.***

| Mediation Estimates | | | | | | | | | | | | | |
| --- | --- | --- | --- | --- | --- | --- | --- | --- | --- | --- | --- | --- | --- |
|  | | | | | | **95% Confidence Interval** | | | |  | | | |
| **Effect** | | **Estimate** | | **SE** | | **Lower** | | **Upper** | | **Z** | | **p** | |
| Indirect |  | 0.0205 |  | 0.0783 |  | -0.133 |  | 0.1740 |  | 0.262 |  | 0.793 |  |
| Direct |  | -0.0795 |  | 0.0906 |  | -0.257 |  | 0.0981 |  | -0.878 |  | 0.380 |  |
| Total |  | -0.0590 |  | 0.1197 |  | -0.294 |  | 0.1757 |  | -0.493 |  | 0.622 |  |
|  | | | | | | | | | | | | | |

| Path Estimates | | | | | | | | | | | | | | | | | |
| --- | --- | --- | --- | --- | --- | --- | --- | --- | --- | --- | --- | --- | --- | --- | --- | --- | --- |
|  | | | | | | | | | | **95% Confidence Interval** | | | |  | | | |
|  | |  | |  | | **Estimate** | | **SE** | | **Lower** | | **Upper** | | **Z** | | **p** | |
| Police Effectiveness |  | → |  | Retaliation |  | 0.0337 |  | 0.1284 |  | -0.218 |  | 0.2853 |  | 0.262 |  | 0.793 |  |
| Retaliation |  | → |  | Aggressive |  | 0.6099 |  | 0.0286 |  | 0.554 |  | 0.6659 |  | 21.337 |  | < .001 |  |
| Police Effectiveness |  | → |  | Aggressive |  | -0.0795 |  | 0.0906 |  | -0.257 |  | 0.0981 |  | -0.878 |  | 0.380 |  |
|  | | | | | | | | | | | | | | | | | |

**Hypothesis 2b was not supported.**

***H3b. High economic threat will lead to stronger non-aggressive confrontation through increased endorsement of honor values on family reputation.***

| Mediation Estimates | | | | | | | | | | | | | |
| --- | --- | --- | --- | --- | --- | --- | --- | --- | --- | --- | --- | --- | --- |
|  | | | | | | **95% Confidence Interval** | | | |  | | | |
| **Effect** | | **Estimate** | | **SE** | | **Lower** | | **Upper** | | **Z** | | **p** | |
| Indirect |  | 0.0133 |  | 0.0367 |  | -0.0587 |  | 0.0852 |  | 0.362 |  | 0.718 |  |
| Direct |  | -0.1789 |  | 0.0975 |  | -0.3700 |  | 0.0123 |  | -1.834 |  | 0.067 |  |
| Total |  | -0.1656 |  | 0.1042 |  | -0.3698 |  | 0.0386 |  | -1.589 |  | 0.112 |  |
|  | | | | | | | | | | | | | |

| Path Estimates | | | | | | | | | | | | | | | | | |
| --- | --- | --- | --- | --- | --- | --- | --- | --- | --- | --- | --- | --- | --- | --- | --- | --- | --- |
|  | | | | | | | | | | **95% Confidence Interval** | | | |  | | | |
|  | |  | |  | | **Estimate** | | **SE** | | **Lower** | | **Upper** | | **Z** | | **p** | |
| Financial Threat |  | → |  | Family Reputation |  | 0.0416 |  | 0.1150 |  | -0.184 |  | 0.2670 |  | 0.362 |  | 0.717 |  |
| Family Reputation |  | → |  | Non-Aggressive |  | 0.3190 |  | 0.0343 |  | 0.252 |  | 0.3863 |  | 9.289 |  | < .001 |  |
| Financial Threat |  | → |  | Non-Aggressive |  | -0.1789 |  | 0.0975 |  | -0.370 |  | 0.0123 |  | -1.834 |  | 0.067 |  |
|  | | | | | | | | | | | | | | | | | |

**Hypothesis 3b was not supported.**

***H4b. Low police effectiveness will lead stronger non-aggressive confrontation through increased endorsement of honor values on family reputation.***

| Mediation Estimates | | | | | | | | | | | | | |
| --- | --- | --- | --- | --- | --- | --- | --- | --- | --- | --- | --- | --- | --- |
|  | | | | | | **95% Confidence Interval** | | | |  | | | |
| **Effect** | | **Estimate** | | **SE** | | **Lower** | | **Upper** | | **Z** | | **p** | |
| Indirect |  | 0.0270 |  | 0.0368 |  | -0.0452 |  | 0.0992 |  | 0.732 |  | 0.464 |  |
| Direct |  | -0.1462 |  | 0.0976 |  | -0.3375 |  | 0.0452 |  | -1.497 |  | 0.134 |  |
| Total |  | -0.1192 |  | 0.1043 |  | -0.3236 |  | 0.0851 |  | -1.144 |  | 0.253 |  |
|  | | | | | | | | | | | | | |

| Path Estimates | | | | | | | | | | | | | | | | | |
| --- | --- | --- | --- | --- | --- | --- | --- | --- | --- | --- | --- | --- | --- | --- | --- | --- | --- |
|  | | | | | | | | | | **95% Confidence Interval** | | | |  | | | |
|  | |  | |  | | **Estimate** | | **SE** | | **Lower** | | **Upper** | | **Z** | | **p** | |
| Police Effectiveness |  | → |  | Family Reputation |  | 0.0843 |  | 0.1149 |  | -0.141 |  | 0.3095 |  | 0.734 |  | 0.463 |  |
| Family Reputation |  | → |  | Non-Aggressive |  | 0.3196 |  | 0.0344 |  | 0.252 |  | 0.3870 |  | 9.295 |  | < .001 |  |
| Police Effectiveness |  | → |  | Non-Aggressive |  | -0.1462 |  | 0.0976 |  | -0.338 |  | 0.0452 |  | -1.497 |  | 0.134 |  |
|  | | | | | | | | | | | | | | | | | |

**Hypothesis 4b was not supported.**

**Study 3: Explanatory Experimental Findings on Avoidance / Humour**

| Independent Samples T-Test | | | | | | | |
| --- | --- | --- | --- | --- | --- | --- | --- |
|  | |  | | **Statistic** | | **p** | |
| Avoidance / Humour |  | Mann-Whitney U |  | 45769 |  | 0.745 |  |
| Note. Hₐ μ _Low_ ≠ μ _High_ | | | | | | | |
|  | | | | | | | |

| Group Descriptives | | | | | | | | | | | | | |
| --- | --- | --- | --- | --- | --- | --- | --- | --- | --- | --- | --- | --- | --- |
|  | | **Group** | | **N** | | **Mean** | | **Median** | | **SD** | | **SE** | |
| Avoidance / Humour |  | Low Financial Threat |  | 314 |  | 3.55 |  | 3.50 |  | 1.61 |  | 0.0908 |  |
|  | | High Financial Threat |  | 296 |  | 3.49 |  | 3.50 |  | 1.72 |  | 0.100 |  |
|  | | | | | | | | | | | | | |

| Independent Samples T-Test | | | | | | | |
| --- | --- | --- | --- | --- | --- | --- | --- |
|  | |  | | **Statistic** | | **p** | |
| Avoidance / Humour |  | Mann-Whitney U |  | 45285 |  | 0.571 |  |
| Note. Hₐ μ _Low_ ≠ μ _High_ | | | | | | | |
|  | | | | | | | |

| Group Descriptives | | | | | | | | | | | | | |
| --- | --- | --- | --- | --- | --- | --- | --- | --- | --- | --- | --- | --- | --- |
|  | | **Group** | | **N** | | **Mean** | | **Median** | | **SD** | | **SE** | |
| Avoidance / Humour |  | Low Police Effectiveness |  | 306 |  | 3.56 |  | 4.00 |  | 1.69 |  | 0.0965 |  |
|  | | High Police Effectiveness |  | 304 |  | 3.48 |  | 3.50 |  | 1.64 |  | 0.0942 |  |
|  | | | | | | | | | | | | | |
